# Mechanistic and preclinical evaluation of SIRT3 as a therapeutic target in melanoma

**DOI:** 10.1080/29944376.2026.2656032

**Published:** 2026-04-14

**Authors:** Karla B. Anaya Aldrete, Mary A. Ndiaye, Glorimar Guzmán-Pérez, Gabriella R. Zaemisch, Chandra K. Singh, Gagan Chhabra, Nihal Ahmad

**Affiliations:** a Department of Dermatology, University of Wisconsin, Madison, Wisconsin, USA;; b William S. Middleton Veterans’ Hospital, Madison, Wisconsin, USA

**Keywords:** SIRT3, PDX, siRNA, 4′-bromo-resveratrol, melanoma

## Abstract

Despite advances in targeted inhibitors and immunotherapies, melanoma remains one of the deadliest skin cancers due to high metastatic potential and resistance to current therapies. This underscores a critical need for new, mechanism-based treatment strategies. Sirtuins, a family of class III histone deacetylases, have been implicated by our lab and others in numerous cellular functions, diseases, and cancers including melanoma. Here, we sought to extend our findings on the pro-proliferative role of SIRT3 by: (i) defining downstream mechanisms using PCR array and NanoString gene-expression profiling on CRISPR/Cas9-mediated SIRT3 knockout (KO) in melanoma cells; (ii) assessing effects of siRNA against SIRT3 in patient-derived xenograft (PDX) and Braf^V600E^/Pten^NULL^ melanoma models; and (iii) evaluating efficacy of SIRT3 inhibitor 4′-Bromo-resveratrol (4′-BR) in PDXes. SIRT3 KO significantly reduced growth and colony formation in A375 and G361 melanoma cells. PCR array and NanoString analysis revealed modulation of key cancer-related signaling molecules. *In vivo*, siRNA-mediated SIRT3 inhibition in melanoma PDXes and Braf^V600E^/Pten^NULL^ mice trended toward reduced tumor growth. However, dual SIRT1/SIRT3 inhibition with 4′-BR (50 mg/kg, *ip*, 2x/week) significantly decreased tumor volume and weight in melanoma PDXes. Overall, our data suggests that targeting SIRT1 and SIRT3 together could offer greater therapeutic benefit and should be validated and optimized.

## Introduction

Melanoma, arising from pigment-producing cells (melanocytes) in the skin, is one of the deadliest skin cancers due to its tendency to metastasize and its resistance to existing therapies. In the United States, in 2026, approximately 112,000 new cases of melanoma are expected to be diagnosed and approximately 8,510 fatalities are expected due to this neoplasm [1]. Though surgical resection is generally successful when melanoma is detected early, more aggressive treatment regimens including immunotherapies as well as targeted therapies (e.g., BRAF and MEK inhibitors) are required for later stage neoplasms [2]. However, many patients develop resistance to the treatments and have recurrence of their melanoma. Therefore, it is imperative to identify novel targets that could be used alone or in combination with existing therapeutics, for an efficient management of this neoplasm.

The mammalian sirtuins are a family of seven class III histone deacetylases (SIRT1-SIRT7) that have been found to play important roles in cell cycle and chromatin regulation, DNA repair, metabolism, aging, and the development and progression of multiple cancers, including melanoma [3–6]. Published research from our lab has shown that several sirtuin family members, including SIRT1 and SIRT3, have altered levels in melanoma [7,8]. SIRT1 is the founding and most well-known member of this family and can be localized to the nucleus or the cytoplasm [9]. Like several other sirtuins, SIRT1 appears to have a cell-type specific effect on cancers [3]. In melanoma, its inhibition has demonstrated an anti-cancer role by causing cell cycle arrest, senescence like phenotype, impairing lamellipodium extension and cell migration, and a direct interaction with p53 acetylation [7,10–15]. Similarly, SIRT3, a mitochondrial sirtuin, has been found to have context-specific effects on cancer and is found to be overexpressed in melanoma tissues and cell lines [8,16]. Like SIRT1, inhibition of SIRT3 has been shown to result in anti-proliferative effects, cell cycle arrest, induction of senescence and altered mitochondrial metabolism, as well as remarkable tumor growth reduction in a mouse xenograft model [8,17]. In a 2019 study published by our lab, we found that small molecule inhibition of SIRT3 via 4′-bromo-resveratrol (4′-BR, which also inhibits SIRT1) had a significant antiproliferative response which was tied to increased apoptosis markers and metabolic reprogramming effects [18]. Additionally, in another previously published study employing the Braf^V600E^/Pten^NULL^ mouse melanoma model, which recapitulates human disease, we have demonstrated that 4′-BR treatment resulted in decreases in tumor growth, metastasis to the lungs, and modulation of key markers of melanoma progression [19].

This study was undertaken to validate our findings on pro-proliferative effects of SIRT3 in additional model systems by determining i) the downstream mechanism of SIRT3 using CRISPR/Cas9-mediated knockout in melanoma cells *in vitro*, coupled with a PCR array and NanoString gene expression profiling, ii) the effect of small interfering RNA (siRNA)-mediated knockout of SIRT3 on melanoma tumor growth in patient-derived xenograft (PDX) as well as in Braf^V600E^/Pten^NULL^ melanoma models, and iii) the effect of 4′-BR on melanoma PDXes.

## Methods

### Cell culture and SIRT3 knockout cell generation

CRISPR/Cas9-mediated A375 and G361 SIRT3 knockout (KO) cell pools and their wild-type (WT) counter-parts were purchased from Synthego. The guide sequence used for A375 cells was CUGAAGUCUGGAAUGCCACU, while the sequence for G361 cells was UUUUCUGUCAGUAUUAAAGG. For A375 cells, DMEM media (Corning #10-013-CV) was used while for G361 cells, 5 A McCoy’s (Corning #10-050-CV) media was used and both media were supplemented with 10% FBS (Sigma #F2442). Cells were grown using standard cell culture conditions (37 °C, 5% CO_2_, humidified chamber) and were authenticated and tested for mycoplasma at regular intervals using Lonza MycoAlert PLUS kit (#LT07-710). To isolate and grow single clones, cell pools for A375 and G361 were plated in 96-well plates at an average of 0.5 cells/well and allowed to grow until a single, discrete colony could be seen in the well. Wells with single colonies were expanded to determine SIRT3 protein expression, and two SIRT3 CRISPR KO clones (A and B) were selected from each cell line for use in growth experiments. For PCR array and Nanostring nCounter assays that were performed, clone B was used for A375 and clone A was used for G361.

### Protein isolation, quantification, and immunoassay via ProteinSimple

Protein was isolated from cell pellets with 1X RIPA lysis buffer (Millipore #20–188) supplemented with phenylmethylsulfonyl fluoride (PMSF, Amresco #0754) and protease inhibitor cocktail (Thermo Scientific/Pierce #78444) supplements and quantified using Pierce BCA Protein assay kit (Thermo Scientific/Pierce #23225) per manufacturer’s protocol using a BioTek Synergy H1 plate reader. Protein expression was determined using the Jess Simple Western automated protein capillary electrophoresis system (ProteinSimple) via manufacturer’s protocol. Briefly, protein lysates, antibodies, and other reagents were pipetted into designated wells in the ProteinSimple microplate (12–230 kDa) and run using default settings. Primary antibody (SIRT3, Cell Signaling #2627) was optimized accordingly before selecting the appropriate conditions for running the experimental immunoassay (0.5 μg/μl lysate concentration; 1:100 antibody dilution). Quantitative data analysis was done via Compass software (ProteinSimple) where peak area value of each sample was normalized with the total capillary area of the respective sample using the Total Protein Assay. Significance was determined using Ordinary One-Way ANOVA via GraphPad Software (version 10.4.1).

### RealTime-Glo MT cell viability assay

RealTime-Glo MT cell viability assay (Promega #G9713) was performed per the manufacturer’s protocol. Briefly, cells were counted and added (A375 = 750 cells per well, G361 = 1000 cells per well) in replicates in a 96-well plate with RealTime-Glo reagents. A BioTek Synergy H1 Multimode plate reader was used to measure luminescence at the indicated times, and individual well reads were normalized back to the initial 1 hr timepoint reading to account for well-to-well variation. Significance was determined using Ordinary One-Way ANOVA via GraphPad Software (version 10.4.1).

### Clonogenic cell survival assay

Cells were added (A375 = 250 cells per well, G361 = 750 cells per well) into 3 wells of 6-well plates and allowed to grow for 10–14 days under standard cell culture conditions with media change every 3–4 days. After discrete colonies could be distinguished, cells were fixed in methanol (Fisher #A412) and stained using crystal violet (Fisher Scientific #C581; 0.1% w/v, 1:1 ddH_2_O:MeOH) at room temperature for 5–10 min before destaining with 1X PBS until background was clear and left to air dry overnight. The following day, digital images were taken.

### RNA isolation and quantification, cDNA preparation, and RT-qPCR

RNA was isolated using RNeasy Plus Mini Kit (Qiagen #74134) according to manufacturer’s protocols and quantified using the Take3 plate on the BioTek Synergy H1 Multimode plate reader. cDNA was prepared using MMLV reverse transcriptase (MMLV-RT, Promega #M1705) and Oligo dT primers (Promega #C1101) and used for qPCR and PCR Arrays. Samples were tested for gDNA contamination using PrimePCR DNA contamination control (Bio-Rad, cat #10025352) following manufacturer’s instructions using iTaq Universal SYBR Green Supermix (BioRad #1725124) on the CFX96 Touch Real-Time PCR Detection System (BioRad).

### RT2 profiler PCR array (Human cancer PathwayFinder)

The Human Cancer PathwayFinder PCR array (Qiagen #PAHS-033ZA) was performed per manufacturer’s protocols on cDNA from A375 wildtype and SIRT3 CRISPR KO cells. Resulting Ct values for each gene/sample were uploaded to the Qiagen GeneGlobe Data Analysis Center and analyzed using GAPDH and ACTB as housekeeping genes. The data analysis web portal calculated fold-change using the ΔΔCT method and those genes showing ≥ 1.5-fold change and p value < 0.05 were considered significantly modulated genes. Significantly modulated genes are provided in Supplemental Table 1 and were uploaded into Ingenuity Pathway Analysis (IPA) software (Content version: 153384343), which was used to predict altered gene interactions, molecular pathways, and cellular functions.

### NanoString Human Tumor Signaling 360 Panel and network analysis

The nCounter Human Tumor Signaling 360 Panel (NanoString #XT-CSO-H-TS360-12) was performed at the University of Wisconsin Translational Research Initiatives in Pathology (TRIP) lab using the nCounter MAX system following manufacturer’s protocols. RNA was isolated from A375 and G361 WT and SIRT3 KO cells in three biological and technical replicates as described above, and the expression of each gene was normalized to endogenous genes. The ROSALIND data analysis platform from NanoString was used to carry out QC report, data normalization, and differential gene expression analysis. Additionally, the functional pathways as well as the hallmarks of cancer affected by SIRT3 inhibition in melanoma were identified. Genes with ≥ 1.5-fold change and p value < 0.05 were considered significantly modulated and were used to perform gene network analyses for both A375 and G361 (Supplemental Tables 2 and 3, respectively). We used the STRING database (version 11.5, https://string-db.org) to predict gene interactions based on literature evidence. Among the specifications selected to create gene networks, the minimum required interaction score was set at a confidence level 0.4, inflation parameter to visualize clusters was set at 1.5 for the Markov Cluster algorithm, and network edges were defined as type of interaction between the genes. In order to better visualize the gene networks, they were transferred from STRING database to Cytoscape (version 3.9.1), and final edits were done with Adobe Illustrator.

### In vivo experiments

All animal studies were approved by the William S. Middleton Memorial Veterans Hospital Institutional Animal Care and Use Committee. Consultation with a statistician and power analysis helped determine our sample size, which is expected to provide 80–95% power, considering a common standard variation of 0.25 and significance of 0.025–0.05. All animals were acclimated per veterinary recommendations in the Animal Resource Facility before beginning experiments. Only animals that could not make it to the end of the study due to health concerns (unrelated to experimental conditions) were excluded.

The patient-derived xenograft mouse model Mel-PDX-01 was established in our lab from melanoma tissue resected during standard of care surgery at the William S. Middleton VA Medical Center from a properly consented male individual. All procedures were conducted in accordance with the principles stated in the Declaration of Helsinki and tissue was collected and used with approval from the University of Wisconsin Institutional Review Board (approval #2016–1196) and written informed consent from participating patients. To develop Mel-PDX-01, excess tissue was diced into a fine slurry and injected subcutaneously on the flanks of immunocompromised mice. Established tumors were resected and used to establish subsequent passages/tumors, with experimental tumors engrafted into female NIH III HO mice (Charles River strain #201; n = 7–9 per group specified by group later). TM01137 is a PDX made from melanoma resected from a female patient and was purchased from The Jackson Laboratories and propagated using the same methods and engrafted from and into NSG mice (NOD.Cg-Prkdc^scid^ Il2rg^tm1Wjl^/SzJ, Jackson Laboratories strain #005557) (n = 5–8 per group; specified by group later). All experimental mice were naïve and not used for previous procedures. Mel-PDX-01 tumors were measured using digital vernier calipers, with tumor volume calculated using the formula π/6*l*w*h, and TM01137 tumors were measured using the Peira TM900 3D measurement device and tumor volume determined using its algorithm. After engraftment, animals were monitored weekly for tumor volume and randomly enrolled in the study once tumor reached at least 50 mm3. Mice were then randomized using Microsoft Excel into four groups (siNTC, siSIRT3, DMSO, 4′-BR) to ensure similar average starting volume in each group and dosing regimen was started as defined below.

The B6.Cg-Tg(Tyr-cre/ERT2)13Bos Braf^tm1Mmcm^ Pten^tm1Hwu^/BosJ (Braf^V600E^/Pten^NULL^) mouse model [20] was purchased from The Jackson Laboratories (strain #013590) and breeding was performed at the University of Wisconsin by the Biomedical Research Model Services core. Experimental mice for this strain had the genotype Hemizygous for Tg(Tyr-cre/ERT2)13Bos, Heterozygous for Braf < tm1Mmcm>, and Homozygous for Pten < tm1Hwu>, and 11–12 mice per group were used for experiments as outlined later. All experimental mice were naïve and not used for previous procedures. Digital vernier calipers were used to measure Braf^V600E^/Pten^NULL^ tumors at the indicated timepoints, with tumor volume calculated using π/6*l*w*h. Tumors were induced using 4-hydroxytamoxifen (4-HT; Cayman Chemical #14854) similar to our previously published work [19], with the exceptions that the 4-HT was dissolved in 100% ethanol before application and tumor induction was allowed to proceed for 4 weeks until visible tumors were present. Mice were randomized at 4 weeks post-tumor induction using Excel into two groups (siNTC, siSIRT3) to ensure similar average starting volume in each group and dosing regimen was started as defined below.

When performing tumor volume measurements, the investigator doing the measurements (KAA, MN, or GZ) was different than the investigator recording the measurements (any other coauthor) to allow for partial blinding. To ensure consistency, the same investigator performed all measurements of each experiment across the timepoints. All investigators participated in euthanasia and collection timepoints. Euthanasia was performed using carbon dioxide per the American Veterinary Medical Association (AVMA) guidelines (2020 Edition). At the end of the experiments, tumors were excised and weighed. The following parameters were assessed throughout and at the end of the experiment: tumor volume, final tumor weight, and body weight, as well as visual lung metastases (in the Braf^V600E^/Pten^NULL^ and TM01137 models). Significance for body weight and tumor weight were determined using t-tests, and significance for tumor volumes was determined using t-tests of area under the curve (AUC) as calculated via GraphPad Software (version 10.4.1).

### siRNA and 4′-BR in vivo treatments

siSTABLE siRNA was obtained from Dharmacon. For the siSIRT3 treatments, the four siRNA were individually solubilized and then combined in equal amounts to deliver 100 μg total pooled siRNA in 20 μL per mouse per injection. This was combined with 16 μL of InvivoJET-PEI (Polyplus/Sartorius #201–50 G) and sterile water and glucose per manufacturer’s instructions to a final volume of 200 μL per mouse and injected intraperitoneally twice per week. The control for the siSIRT3 experiments was non-targeting siRNA (siNTC) and was administered using the same process. The sequences for human (PDX tissues) and mouse (Braf^V600E^/Pten^NULL^) are outlined in Supplemental Table 4, with siNTC being the same for both mouse and human. 4′-Bromo-resveratrol (4′-BR) was obtained from Aobious (cat# AOB2381) and solubilized in DMSO/PEG (control; Sigma #D1435/Spectrum #PO108), filter sterilized, and injected in a final volume of 50 μL per mouse administered intraperitoneally twice per week.

### Quantification of lung metastasis

At the termination of experiment, the mice were euthanized, lungs were resected and fixed in Fekete’s solution [21]. Using a dissecting microscope, visible dark-colored lesions (metastases) from the TM01137 and Braf^V600E^/Pten^NULL^ models were observed on the external surfaces of the lungs and counted. Mel-PDX-01 is non-melanotic, so was unable to be counted. Significance was determined using t-test via GraphPad Software (version 10.4.1).

## Results & discussion

### CRISPR/Cas9-mediated SIRT3 knockout (KO) in human melanoma cells significantly reduces growth and long-term proliferation potential

**Figure 1.**
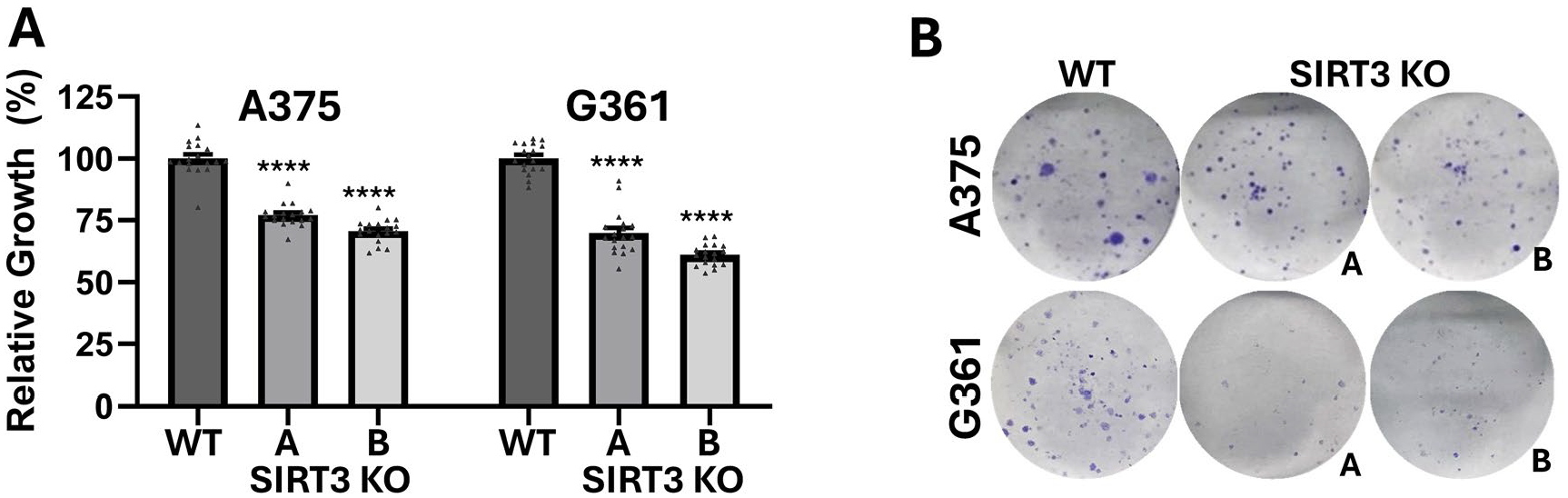
CRISPR/Cas9-mediated SIRT3 knockout (KO) reduces cell growth and clonogenic survival in human melanoma cells. (A) Relative growth at 48 hrs after seeding cells using RealTime Glo cell viability assay and (B) clonogenic survival in A375 and G361 wild-type (WT) and two SIRT3 KO clones (clones A and B). For RealTime Glo, data is normalized to 1 hr baseline reading and presented as mean values ± SEM. Each set is representative of biological and technical triplicate experiments. Statistical significance was determined by Ordinary one-way ANOVA using GraphPad Prism software (****p ≤ 0.0001). For clonogenic survival assay, representative images of each cell group are used.

Previously, we have demonstrated that SIRT3 is overexpressed in melanoma and its inhibition using shRNA or 4′-BR (a dual SIRT1/3 inhibitor) resulted in significant anti-proliferative responses against melanoma *in vitro* and *in vivo* [8,17–19]. To further validate the role of SIRT3 in melanoma and the effects of SIRT3 inhibition in melanoma cells in vitro, we generated CRISPR/Cas9-mediated SIRT3 knockout (KO) A375 and G361 single cell clones with significantly reduced SIRT3 protein levels (Supplemental Figure 1A). We found significant reduction in growth beginning at 48 hours post-plating in the stable clones (Figure 1(A)), along with significant reduction in colony formation abilities in the SIRT3 KO cells (Figure 1(B)). This validated our previous work suggesting that SIRT3 has pro-proliferative functions in melanoma [8,17–19].

### CRISPR/Cas9-mediated SIRT3 knockout (KO) in human melanoma cells significantly impacts traditional cancer pathways

**Figure 2.**
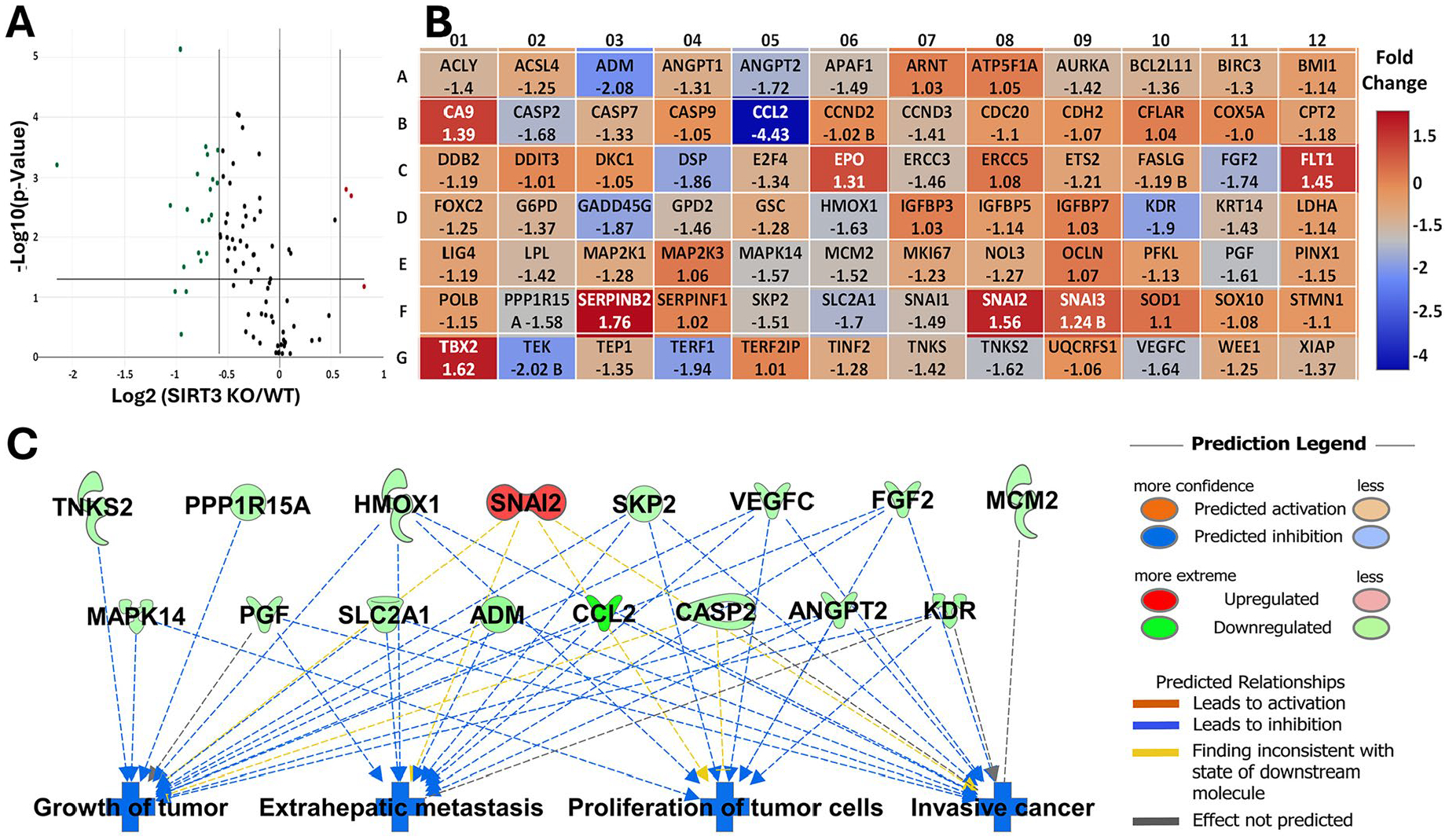
CRISPR/Cas9-mediated SIRT3 knockout (KO) analysis by PCR array finds modulation in key cancer pathways in A375 human melanoma cells. (A) Significantly modulated genes (p ≤ 0.05 and fold change was ≥ 1.5 in SIRT3 KO clone B versus WT A375 cells) were identified using the RT2 Human Cancer PathwayFinder PCR Array and data are shown as a volcano plot. (B) Heat map and Fold Change of individual genes in SIRT3 KO versus WT A375 cells. (C) IPA analysis was used to evaluate up- and down-stream cancer-related genes/pathways after SIRT3 KO in A375 cells.

To further elucidate the mechanism of SIRT3 in melanoma, we used the RT2 Profiler Human Cancer Pathway Finder PCR array, which allows for the analysis of 84 cancer-related genes involved in different cellular processes (i.e., angiogenesis, apoptosis, cell cycle, cellular senescence, DNA damage and repair, epithelial-to-mesenchymal transition (EMT), hypoxia signaling, metabolism, and telomeres and telomerase). SIRT3 KO in A375 cells significantly modulated (≥1.5-fold change and p < 0.05) 18 genes, with 16 downregulated and 2 upregulated (Figure 2(A,B)). Using Ingenuity Pathway Analysis (IPA) software, a commercial bioinformatics platform from Qiagen that can help interpret high-throughput omics data, we analyzed predicted gene networks and interactions paired with potential up- and down- stream targets. Interestingly, IPA predicted that multiple genes and pathways were influenced by SIRT3 KO, leading to inhibition of many cancer-related pathways, including cell proliferation, tumor growth, and metastasis (Figure 2(C)). This supports our previous study with shRNA-mediated SIRT3 knockdown in SK-MEL-2 melanoma cells, underscoring the potential impact of SIRT3 in melanoma [17]. Further, IPA exploration of the gene/pathway network showed the connection of SIRT3 modulated genes along with several melanoma-associated genes (Supplemental Figure 1B).

**Figure 3.**
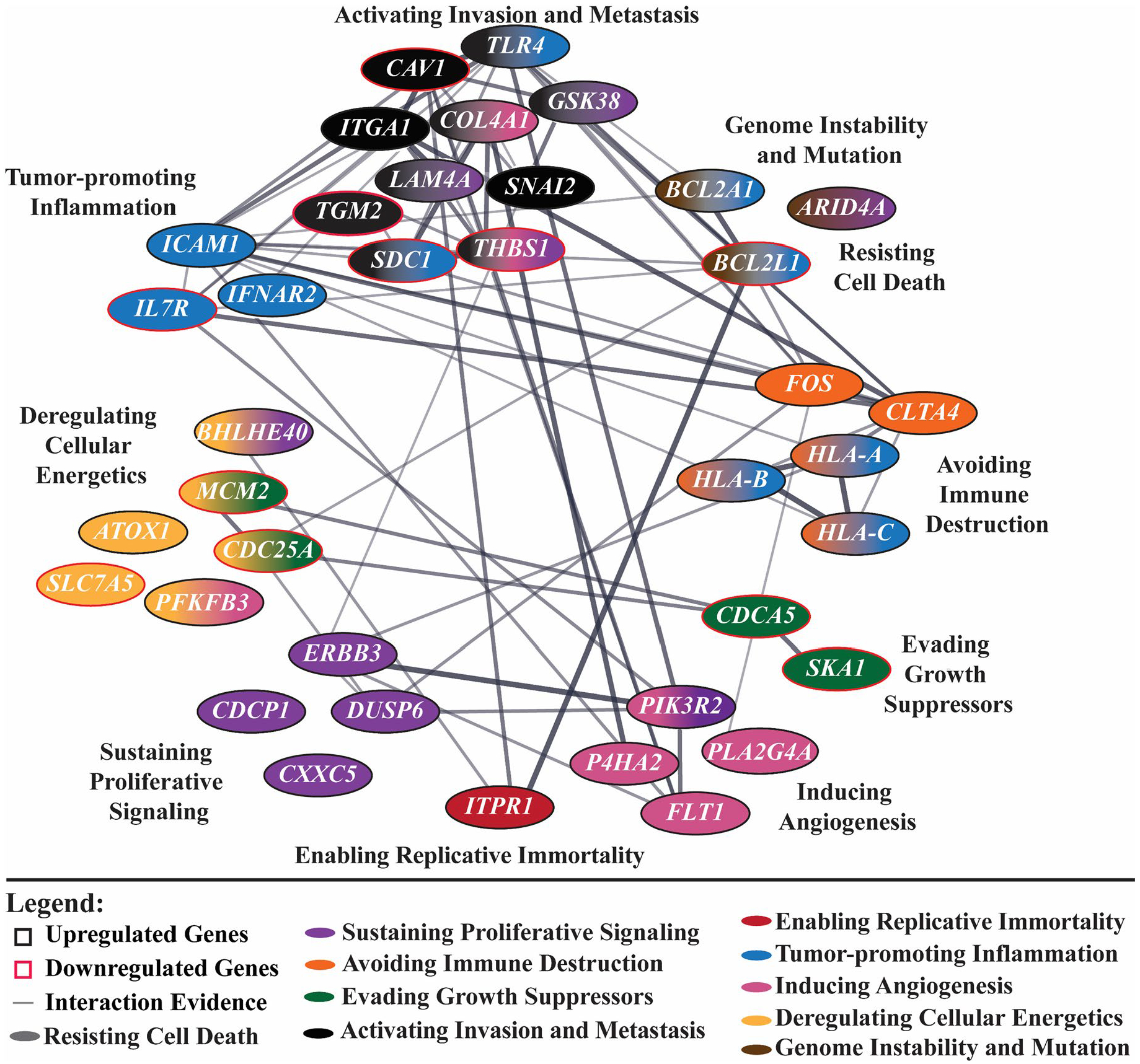
CRISPR/Cas9-mediated SIRT3 knockout (KO) analysis by NanoString nCounter Tumor Signaling Panel uncovers changes in key cancer hallmark-associated genes. Significantly modulated genes (p ≤ 0.05 and fold change ≥ 1.5 in A375 SIRT3 KO cells vs WT) from NanoString nCounter Human Tumor Signaling 360 Panel Analysis resulted in modulation of genes related to cancer hallmarks in SIRT3 KO A375 cells. Hallmarks of cancer include activating invasion and metastasis (black), sustaining proliferative signaling (purple), resisting cell death (grey), avoiding immune destruction (orange), evading growth suppressors (green), enabling replicative immortality (maroon), tumor-promoting inflammation (blue), genome instability & mutation (brown), inducing angiogenesis (pink), and deregulating cellular energetics (yellow). Upregulated genes have black outlines while downregulated genes have red outlines.

To further investigate the mechanistic effects of SIRT3 KO on cancer-related signaling pathways, we performed NanoString nCounter Human Tumor Signaling 360 Panel analysis. This enabled us to assess the impact of SIRT3 inhibition across a broader set of 760 genes involved in tumor signaling networks and established hallmarks of cancer. As listed in Supplemental Table 2, 38 genes were significantly modulated (11 genes were downregulated and 27 upregulated) in A375 cells. G361 cells had 10 differentially expressed genes (2 genes were downregulated and 9 upregulated) (Supplemental Table 3). Networks with the differentially expressed genes in A375 and G361 SIRT3 KO cells were created using STRING network and Cytoscape and are grouped by some commonalities in terms of cancer hallmarks (Figure 3, Supplemental Figure 1C). Similar to the PCR array data, the trend observed in the NanoString data had both tumor promoting and tumor suppressing implications. For example, in A375 cells, *BCL2L1, CAV1, CDC25A, CDCA5, IL7R, MCM2, SDC1, SLC7A5, TGM2*, and *THBS1* were downregulated after SIRT3 KO and have previously been found to have oncogenic-like properties in melanoma [22–32]. Within the genes that were upregulated after SIRT3 inhibition in A375 cells, we found *DUSP6, FOS, GSK3B, HLA-A, HLA-B, HLA-C, ICAM, ITGA1*, and *TLR4*, which have anti-melanoma effects [33–40]. However, most of these genes were immune-related genes, suggesting a careful evaluation of the effects of SIRT3 inhibition in the presence of an active immune system in the tumor microenvironment. Connections between the differentially expressed genes in A375 cells are presented in Figure 3. Less genes were found to be significantly modulated after CRISPR/Cas9-mediated SIRT3 KO in G361 melanoma cells, including 8 upregulated and 2 downregulated (Supplemental Table 3). Out of these genes, upregulation of *IL7*, and *OAS1*, and downregulation of *ASH1L* and *NUF2* support the pro-proliferative function of SIRT3 in melanoma [41–44]. However, upregulation of genes such as *KDR, UGDH, TPD52, LDHA, CD44*, and *EPAS1* do not support its pro-proliferative function in melanoma [45–50]. Altogether, molecular profiling of A375 and G361 cells after CRISPR/Cas9-mediated SIRT3 KO identified potential SIRT3-associated genes in melanoma. However, there were discrepancies between the tested cell lines, as is common with cell culture models [51,52]. Although both cell lines are generated from primary human melanomas, they are different melanoma models with heterogeneous genetic backgrounds, growth patterns, and expression profiles. For example, the A375 cell line was generated from a 54 year-old female melanoma patient [53], while G361 was generated from a 31 year-old male [54]. Also, A375 is an amelanotic cell line, while G361 is melanotic. Moreover, A375 cells tend to exhibit faster proliferation, higher glycolytic activity, and greater sensitivity towards inhibition of specific signaling pathways compared to G361 cells [55]. These differences can make drastic differences in gene expression profiles, so while we found discrepancies between the datasets, we wanted to compare the overall trends found. Additionally, there were conflicting findings between the antiproliferative effects observed in these melanoma cells and the melanoma tumor promoting literature-based evidence that some of these genes were associated with. Therefore, further validation studies should be carried out as well as incorporating other cellular pathways (e.g., ROS production, apoptosis, EMT, etc.).

### In vivo SIRT3 inhibition via siRNA has minimal effects on tumor growth

**Figure 4.**
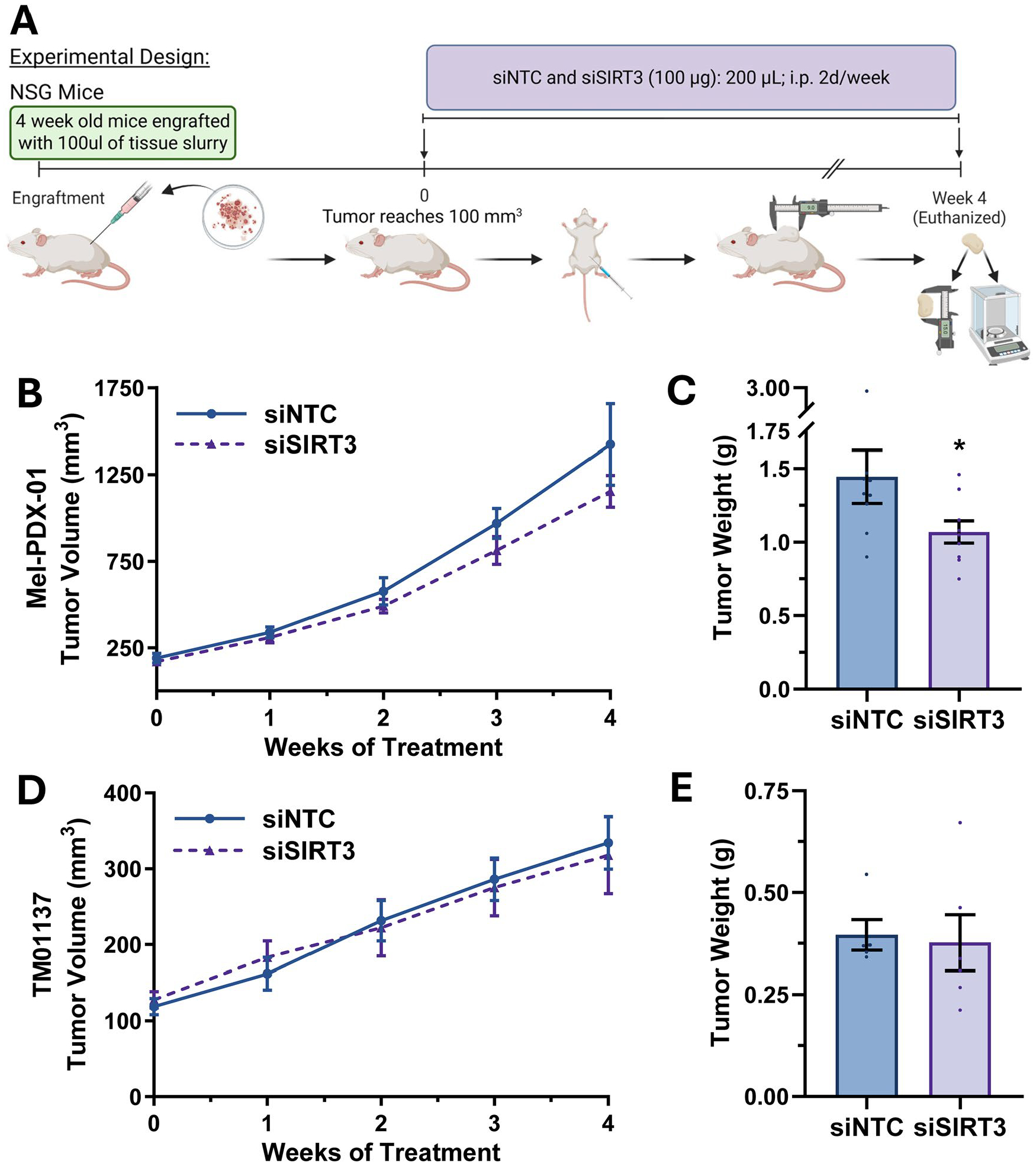
*In vivo* SIRT3 inhibition via siRNA reduces tumor growth in patient-derived xenograft (PDX) mouse models of melanoma. (A) Immunocompromised mice were engrafted with PDX tissues and injected intraperitoneally (i.p) with siRNA against SIRT3 or non-targeting control (NTC) twice weekly for the duration of the study, as depicted in the experimental design. Graphs of tumor volume (B, D) and endpoint tumor weight (C, E) of Mel-PDX-01 (n = 9 per group) and TM01137 (n = 5–6 per group)-engrafted mice are shown, respectively. Statistical significance was determined using GraphPad software and t-test (for area under the curve (AUC) for tumor volume) (*p ≤ 0.05).

In order to validate the pro-proliferative role of SIRT3 in melanoma, we determined the effect of SIRT3 siRNA against melanoma *in vivo*. We utilized two PDX mouse models (Mel-PDX-01 and TM01137) along with the Braf^V600E^/Pten^NULL^ mouse model for our investigation. The experimental design and treatment schedule for the PDX studies are shown in Figure 4(A). Although the reduction in tumor volume did not reach statistical significance, both PDX models exhibited a trend toward decreased tumor burden following siSIRT3 treatment compared with the non-targeting control (siNTC), with the strongest effect observed in the Mel-PDX-01 model (Figure 4(B,D)). This pattern was reflected in final tumor weights (Figure 4(C,E)), where siSIRT3 treatment resulted in a statistically significant reduction in the Mel-PDX-01 cohort. Importantly, siRNA administration did not significantly affect mouse body weight (Supplemental Figure 2A). In addition, quantification of visible lung metastases in the TM01137 model (Mel-PDX-01 was unable to be visibly quantified as it is amelanotic) revealed no significant differences between treatment groups (Supplemental Figure 2B). Taken together with our previous findings, these results lend credence towards the role of SIRT3 as a therapeutic target for melanoma treatment. However, the limited response we found highlights the importance of optimizing delivery and dosing strategies to achieve maximal efficacy.

**Figure 5.**
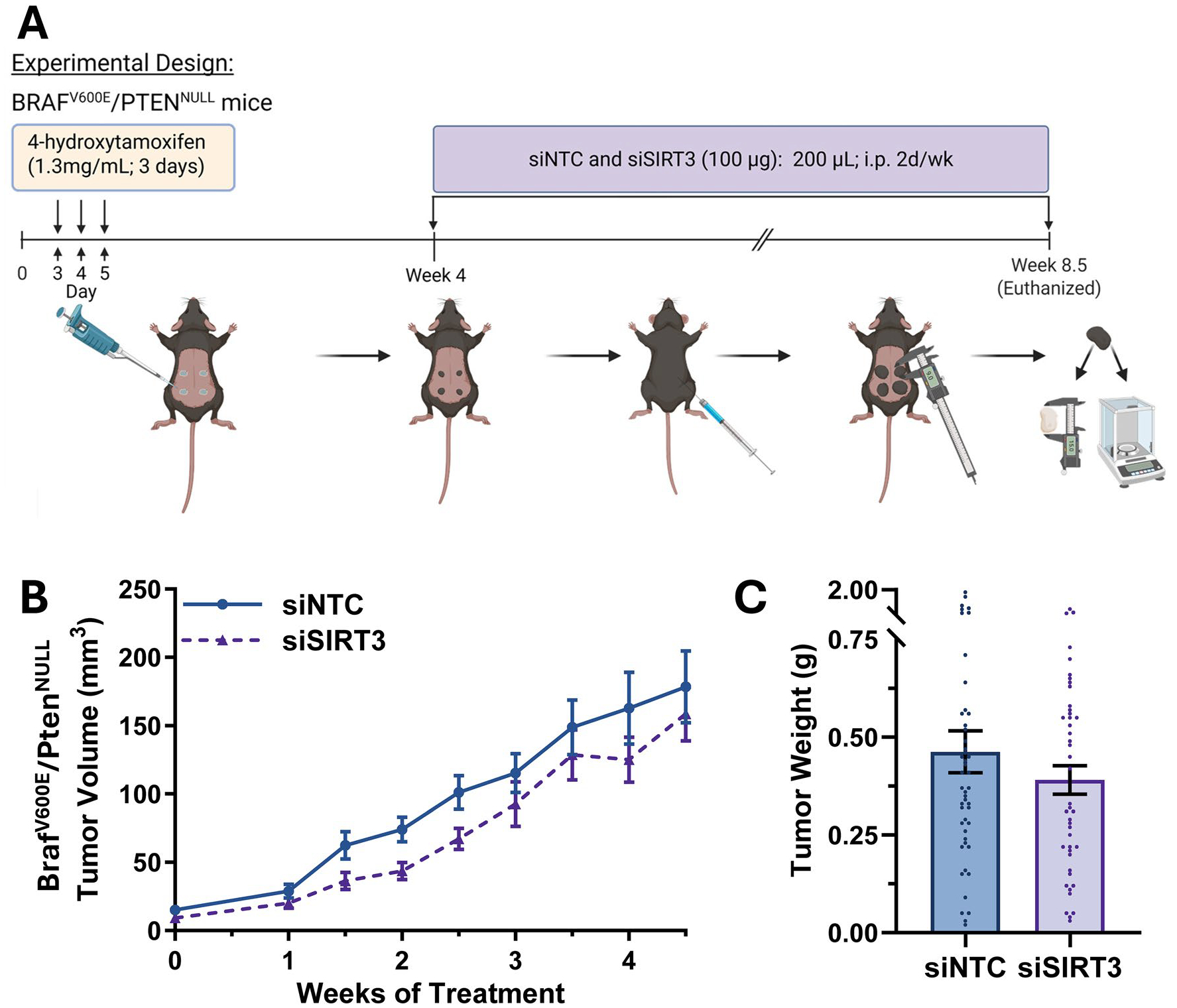
*In vivo* SIRT3 inhibition via siRNA reduces tumor growth in the Braf^V600E^/Pten^NULL^ mouse model of melanoma. (A) Four tumors per mouse were induced on the shaved back of Braf^V600E^/Pten^NULL^ mice (n = 11–12 per group) using 4-hydroxytamoxifen (4-HT) via a standard protocol, and the mice were administered intraperitoneally (i.p.) siSIRT3 or siNTC twice weekly starting at 4 weeks post-induction. Graphs of (B) average individual tumor volume and (C) final individual tumor weight are shown. Statistical significance was determined using GraphPad software and t-test (for area under the curve (AUC) for tumor volume) (*p ≤ 0.05).

Next, we determined the effect of SIRT3 siRNA on melanoma growth in Braf^V600E^/Pten^NULL^ mouse model. This immunocompetent model is highly relevant to human disease, as it develops cutaneous melanoma that progresses and metastasizes in a manner similar to human melanoma [20]. Incorporating this model into our study also enabled us to compare the effects of selectively targeting SIRT3 alone with the dual SIRT1/SIRT3 inhibition strategy we previously reported [19]. The treatment regimen we used for the siRNA administration was similar to the PDX models (Figure 5(A)). Interestingly, we found similar results to the PDX model, with a trend toward reduced tumor growth and final tumor weight (Figure 5(B,C), respectively). Also similar to the PDX models, we found no effect on body weight with the treatment in the Braf^V600E^/Pten^NULL^ mice (Supplemental Figure 2C), along with no significant difference in number of visible lung metastases (Supplemental Figure 2D), although there was a trend towards reduced number of visible nodules. Overall, across all three melanoma mouse models, our SIRT3-targeting siRNA treatment protocol showed a consistent, encouraging trend toward reduced tumor growth; however, these effects did not reach statistical significance. It appears that future studies will require optimization of treatment regimens to achieve clearly effective outcomes.

In our experiments here, we used SIRT3-targeting siRNA in order to mitigate any off-target effects that can complicate results when using targeted therapy agents. However, the use of siRNA comes with its own challenges, especially when aiming for in vivo therapies, including stability, delivery efficiency, dosing administration methods and/or regimens [56–58]. Although we chose our doses and delivery methods based on previously published studies [59,60], it is likely that a different method may be more ideal and provide superior results. Furthermore, our findings suggest that targeting SIRT3 in combination with other targets, such as other pro-proliferative melanoma specific sirtuins, may offer a more effective strategy for melanoma management. This notion is supported by our previous work demonstrating that dual inhibition of SIRT3 and SIRT1 produces significant anti-proliferative and anti-metastatic effects in melanoma models [19]. Through our work and that of others, substantial progress has been made in understanding the roles of multiple sirtuins in melanoma; however, emerging evidence indicates that targeting individual sirtuin family members in isolation may not be sufficient. Instead, combinatorial inhibition of two or more sirtuins may provide a more robust and therapeutically advantageous approach [6].

### Treatment with 4′-BR significantly reduces tumor growth in PDX mouse models

**Figure 6.**
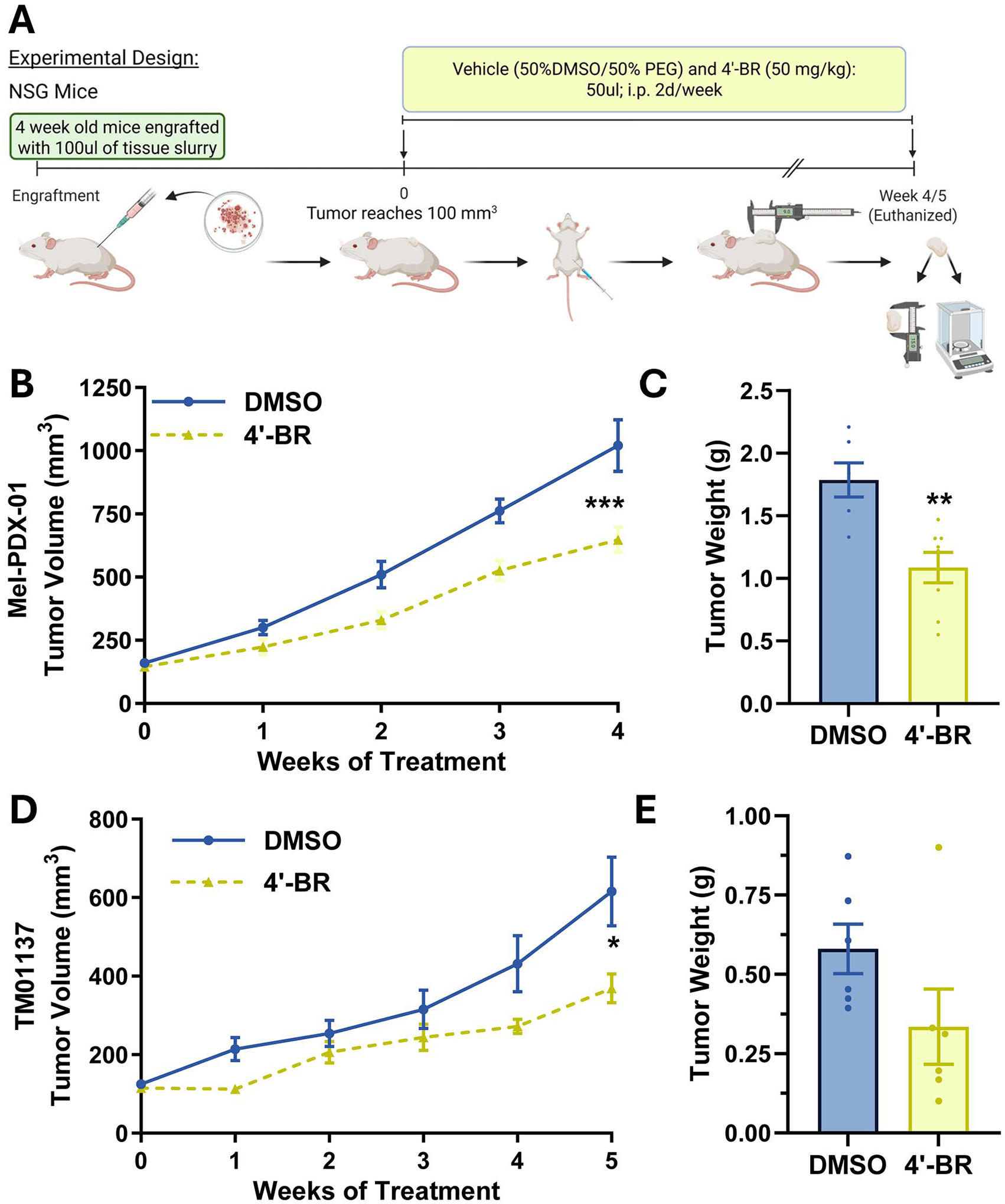
Concomitant SIRT1 and SIRT3 inhibition via 4′-bromo-resveratrol (4′-BR) significantly reduces tumor growth in patient-derived xenograft (PDX) models of melanoma. (A) Immunocompromised mice were engrafted with PDX tissues (n = 7–8 per group) and treated with 4′-BR twice weekly via intraperitoneal (i.p.) injection for the duration of the study, as depicted in the experimental design. Tumor volume (B, D) and final tumor weight (C, E) are shown for the Mel-PDX-01 and TM01137 PDX models, respectively. Statistical significance was determined using GraphPad software and t-tests (for area under the curve (AUC) for tumor volume) (*p ≤ 0.05, **p ≤ 0.01, ***p ≤ 0.001).

As described above, we have earlier demonstrated the anti-proliferative potential of 4′-BR in melanoma utilizing the BRaf^V600E^/PTEN^NULL^ mouse model [19]. While this model is powerful and well-validated and faithfully recapitulates key aspects of human melanoma initiation and progression, it may not fully capture the biological heterogeneity present in human tumors. Consequently, validation in human PDX models would be valuable for demonstrating therapeutic relevance across genetically diverse melanomas and for reducing risk as the approach advances toward clinical translation. Therefore, in our next series of experiments, we employed two PDX models to determine the effects of 4’-BR against melanoma growth. From our previous work [19], we selected a dose of 50 mg/kg 4′-BR twice weekly, administered via intraperitoneal injection (Figure 6(A)). We found a significant decrease in tumor volume in both Mel-PDX-01 and TM01137 PDXes (Figure 6(B,D)). This decrease was also seen in the final tumor weight after 4′-BR treatment, with a significant decrease seen in the Mel-PDX-01, and results close to significance in the TM01137 (not reached due to one large outlier; p value = 0.1150) (Figure 6(C,E)). We found no significant reduction in mouse body weight with 4′-BR treatment (Supplemental Figure 2E). Additionally, since TM01137 is a melanotic tumor, we were able to observe metastatic lesion in the lungs at the time of collections. After quantifying the number of pigmented lesions on the surface of the lungs, a decreased trend in metastatic lesions was observed in the 4′-BR treated lungs, although it did not yield statistical significance (Supplemental Figure 2F). The feasibility of therapeutically targeting sirtuins, including SIRT1 and SIRT3, in cancer is further underscored by a recent study that optimized a mitochondria-targeting SIRT1/2/3 inhibitor (SJ-106C) with improved solubility and potency versus a prior chemotype, and demonstrated in vivo efficacy in Diffuse Large B Cell Lymphoma xenografts at the same dose (50 mg/kg) that we used in this study [61]. However, SJ-106C was administered 5 times/week compared to twice/week 4′-BR in this study. Moreover, the use of PDX models in the current study represents a critical step toward assessing the translational relevance of 4′-BR as a therapeutic agent for melanoma management.

## Conclusion

Our data demonstrated that CRISPR/Cas9-mediated SIRT3 KO resulted in antiproliferative effects in human melanoma cells (reduced cell viability and clonogenic survival). Additionally, we carried out two molecular profiling techniques (PCR Array and NanoString nCounter analysis), where we assessed the effects of CRISPR/Cas9-mediated SIRT3 KO in hundreds of genes involved in tumor signaling as well as cancer-related pathways. Through our studies, we were able to identify potential SIRT3-associated mechanisms in melanoma cells, which can be further explored in future studies. Interestingly, although much of the data agreed between the two cell lines we tested, we saw marked differences between the two cell lines in some of the genes that were significantly modulated, some of which have previously been shown to have opposite effects. This may suggest that additional, non-tested genes and/or cellular contexts may mitigate or alter the effects of SIRT3 in melanoma and should be explored further. Taking this into consideration, SIRT3 seems to support melanoma progression, and simultaneously inhibiting this sirtuin together with other tumor promoting proteins might lead to stronger antimelanoma effects. We directly tested this effect in human melanoma relevant mouse models and found that while targeting SIRT3 alone with siRNA led to a trend towards tumor growth reduction in both PDX and Braf^V600E^/Pten^NULL^ mouse models, it was not enough to reach statistical significance. However, when using 4′-bromo-resveratrol (4′-BR), which inhibits both SIRT3 and SIRT1 activity, we were able to observe a significant tumor growth reduction in two PDX models of melanoma, which matches previous work in the Braf^V600E^/Pten^NULL^ mouse model. Overall, our data suggests that a concomitant inhibition of SIRT1 and SIRT3 (and/or potentially other sirtuins with pro-proliferative roles in melanocytic cells) may be a useful strategy for melanoma management. Additional research is needed to validate our findings and to explore the mechanisms involved.

## Supplementary Material

Supp 1

Supp 2

Supplemental data for this article can be accessed online at https://doi.org/10.1080/29944376.2026.2656032.

## Data Availability

The data that support the findings of this study are available on request from the corresponding author.
