# Supplementary material for: Mechanistic and preclinical evaluation of SIRT3 as a therapeutic target in melanoma
Source: J Derm Oncol. Author manuscript; Available in PMC 2026 May 16. (PMC13178468; doi:10.1080/29944376.2026.2656032)
Supplement: Supp 2 [file NIHMS2168255-supplement-Supp_2.docx]

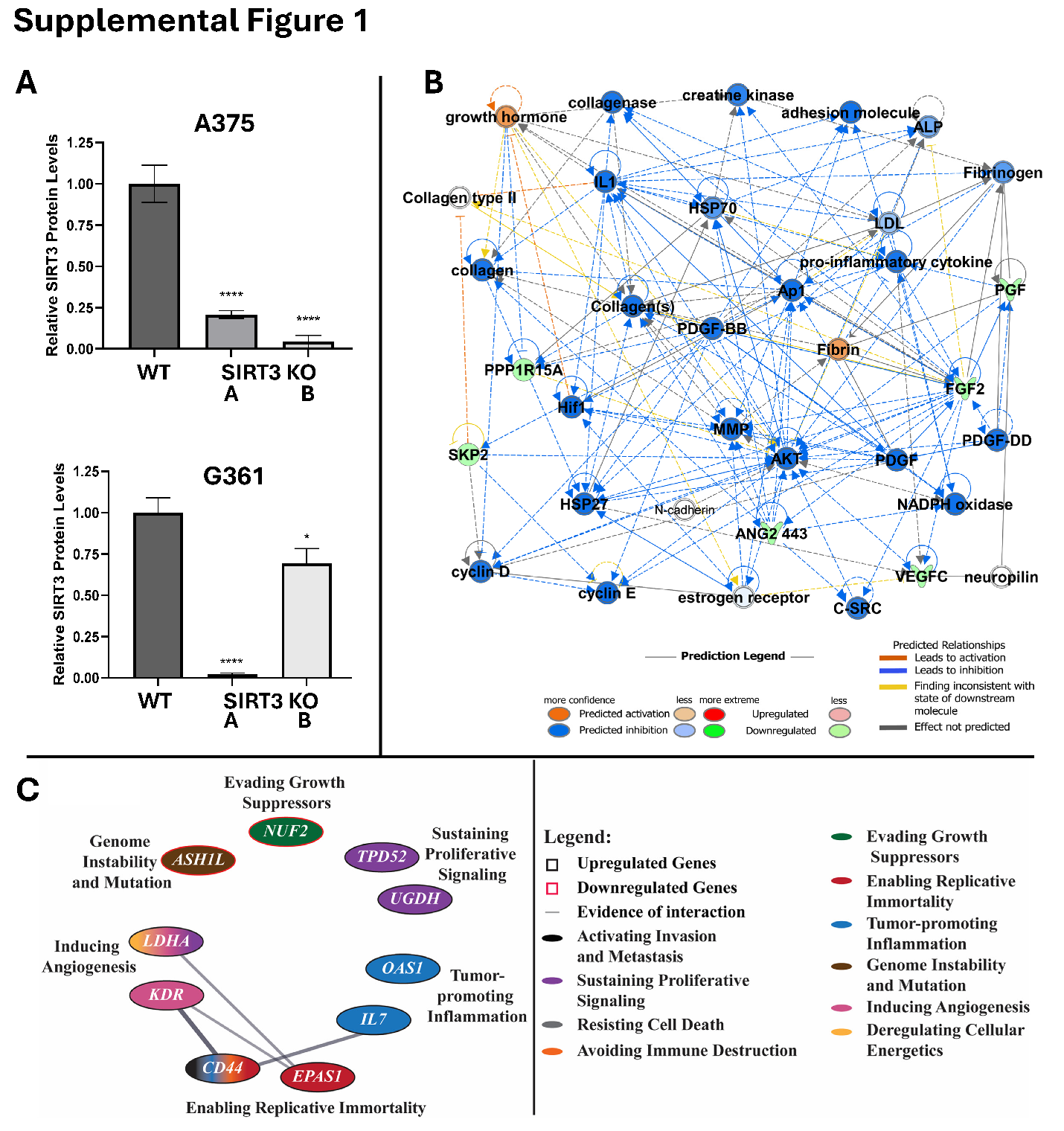


**Supplemental Figure 1. SIRT3 knockout (KO) in human melanoma cells affects SIRT3 protein expression, molecular targets, and cancer pathways.** (A) SIRT3 protein expression levels are significantly reduced in CRISPR/Cas9-mediated SIRT3 KO A375 and G361 clones. Quantitative immunodetection via ProteinSimple Jess was carried out with triplicates of each sample. Lysates were added with equal amounts of protein concentration. Data was normalized with the ProteinSimple Total Protein Assay and is presented as mean protein expression levels ± SEM. Statistical significance was determined by one-way ANOVA (*p ≤ 0.05, ****p < 0.0001). (B) Networks of differentially expressed genes identified in SIRT3 KO A375 cells (clone B was used) by Human Cancer PathwayFinder PCR array and their up- and downstream molecular targets and cancer pathways. Significantly modulated genes were considered when p value < 0.05 and fold change was ≥ 1.4 in SIRT3 KO cells compared to wildtype (WT) cells. (C) Significantly modulated genes (p ≤ 0.05 and fold change ≥ 1.5 in SIRT3 KO cells vs WT) from NanoString Human Tumor Signaling 360 Panel Analysis resulted in modulation of genes related to cancer hallmarks in SIRT3 KO G361 cells (clone A was used). Hallmarks of cancer include activating invasion and metastasis (black), sustaining proliferative signaling (purple), resisting cell death (grey), avoiding immune destruction (orange), evading growth suppressors (green), enabling replicative immortality (maroon), tumor-promoting inflammation (blue), genome instability & mutation (brown), inducing angiogenesis (pink), and deregulating cellular energetics (yellow). Upregulated genes have black outlines while downregulated genes have red outlines.


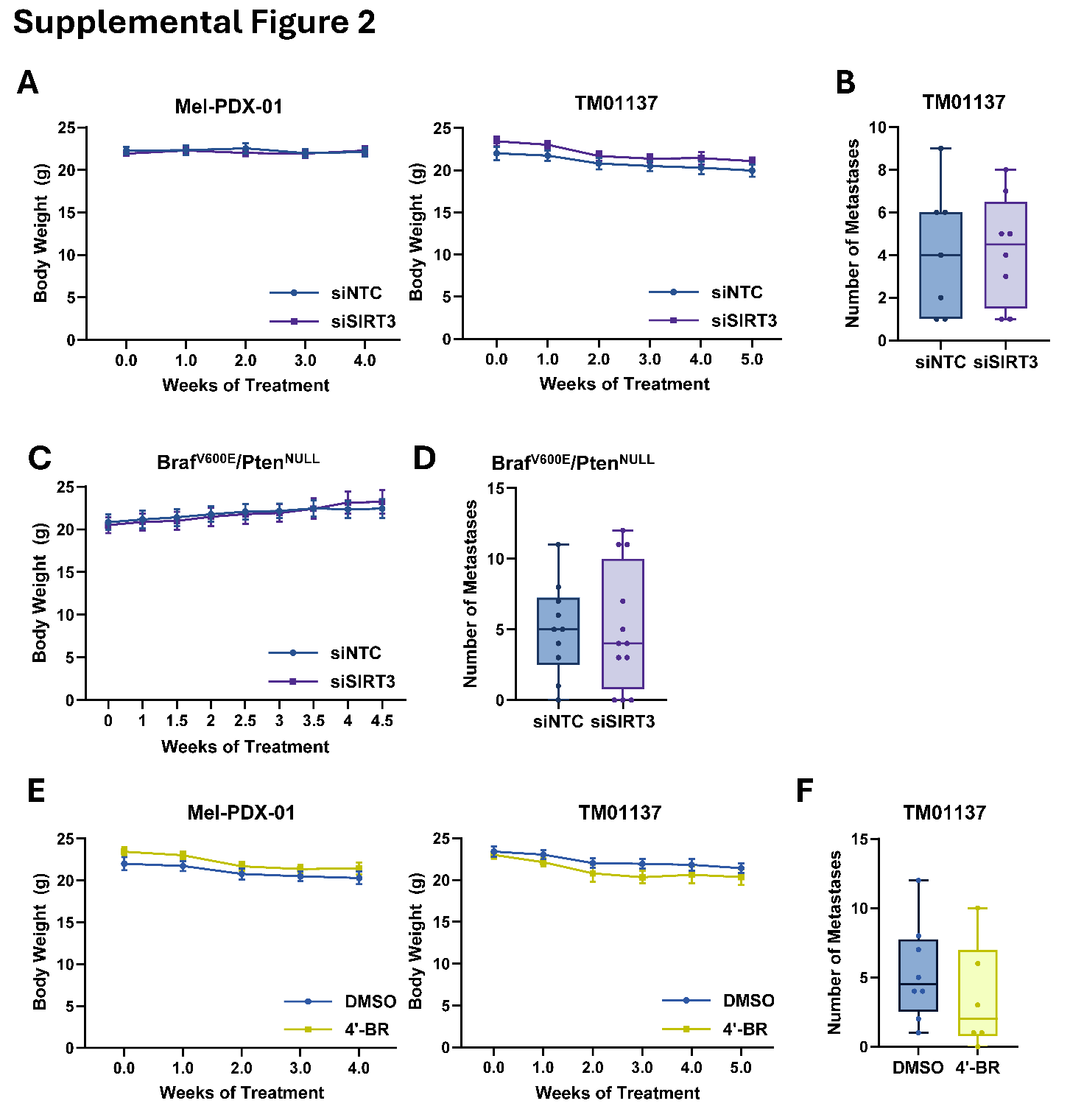


**Supplemental Figure 2. Inhibition of SIRT3 alone via siRNA or in combination with SIRT1 via 4'-Bromo-resveratrol (4'-BR) has no significant effect on body weight and reduces lung metastases in patient-derived xenograft (PDX) and Braf^V600E^/Pten^NULL^ mice.** (A) Body weight measurements of immunocompromised mice engrafted with PDX models (PDX01 or TM01137) throughout siRNA treatment. (B) Visual counts of melanotic lung surface nodules in TM01137‑engrafted mice at the end of the siRNA treatment period. (C) Body weight of Braf^V600E^/Pten^NULL^ mice during siRNA treatment. (D) Visual counting of melanotic surface lung nodules in Braf^V600E^/Pten^NULL^ mice after siRNA treatment conclusion. (E) Body weight of immunocompromised mice engrafted with PDX models (PDX01 or TM01137) during siRNA treatment. (F) Visual counting of melanotic surface lung nodules in TM01137-engrafted mice after siRNA treatment conclusion.

**Supplemental Table 1**. Human Cancer PathwayFinder PCR Array significantly modulated genes in CRISPR/Cas9-mediated SIRT3 KO A375 human melanoma cells.

| Gene | Description | Molecular functions | Fold Change | p value |
| --- | --- | --- | --- | --- |
|  |  |  |  |  |
| *SNAI2* | Snail family transcriptional repressor 2 | EMT | 1.56 | 0.00159 |
| *TBX2* | T-box transcription factor 2 | Cellular Senescence | 1.62 | 0.00204 |
| *SKP2* | S-phase kinase associated protein 2 | Cell Cycle | -1.51 | 0.000351 |
| *MCM2* | Minichromosome maintenance complex component 2 | Cell Cycle | -1.52 | 0.00125 |
| *MAPK14* | Mitogen-activated protein kinase 14 | Cellular Senescence | -1.57 | 0.00108 |
| *PPP1R15A* | Protein phosphatase 1 regulatory subunit 15A | DNA Damage & Repair | -1.58 | 0.00427 |
| *PGF* | Placental growth factor | Angiogenesis | -1.61 | 0.00511 |
| *TNKS2* | Tankyrase 2 | Telomeres & Telomerase | -1.62 | 0.000416 |
| *HMOX1* | Heme oxygenase 1 | Hypoxia Signaling | -1.63 | 0.0187 |
| *VEGFC* | Vascular endothelial growth factor C | Angiogenesis | -1.64 | 0.000308 |
| *CASP2* | Caspase 2 | Apoptosis | -1.68 | 0.00537 |
| *SLC2A1* | Solute carrier family 2 member 1 | Hypoxia Signaling | -1.7 | 0.0249 |
| *ANGPT2* | Angiopoietin 2 | Angiogenesis | -1.72 | 0.0183 |
| *FGF2* | Fibroblast growth factor 2 | Angiogenesis | -1.74 | 0.000883 |
| *DSP* | Desmoplakin | EMT | -1.86 | 0.00345 |
| *KDR* | Kinase insert domain receptor | Angiogenesis | -1.9 | 0.0312 |
| *ADM* | Adrenomedullin | Hypoxia Signaling | -2.08 | 0.00295 |
| *CCL2* | C-C motif chemokine ligand 2 | Angiogenesis | -4.43 | 0.00062 |

Abbreviations: EMT = Epithelial-to-Mesenchymal Transition

**Supplemental Table 2**. NanoString Human Tumor Signaling 360 Panel Analysis of significantly modulated genes after SIRT3 KO in A375 human melanoma cells.

| Gene | Description | Molecular functions | Fold Δ | p value |
| --- | --- | --- | --- | --- |
| *FOS* | FBJ murine osteosarcoma viral oncogene homolog | TCR Signaling | 18.87 | 0.002599 |
| *CTLA4* | Cytotoxic T-lymphocyte-associated protein 4 | T-cell Costimulation; T-cell Exhaustion; TCR Signaling | 15.33 | 0.001469 |
| *ITGA1* | Integrin, alpha 1 | Cell Adhesion & Motility; ECM Remodeling & Metastasis | 4.722 | 0.000573 |
| *BCL2A1* | BCL2-related protein A1 | Apoptosis; Epigenetic & Transcriptional Regulation; NF-kB Signaling | 3.219 | 0.00162 |
| *SNAI2* | Snail family zinc finger 2 | EMT; Hippo Signaling | 3.120 | 0.001392 |
| *HLA-B* | Major histocompatibility complex, class I, B | Antigen Presentation; Interferon Response | 3.094 | 4.14E-05 |
| *BHLHE40* | Basic helix-loop-helix family, member e40 | mTOR Signaling; TGF-beta Signaling | 3.087 | 3.85E-06 |
| *LAMA4* | Laminin, alpha 4 | Cell Adhesion & Motility; ECM Remodeling & Metastasis; MET Signaling | 2.940 | 0.000467 |
| *PLA2G4A* | Phospholipase A2, group IVA (cytosolic, calcium-dependent) | VEGF Signaling | 2.890 | 0.000362 |
| *HLA-C* | Major histocompatibility complex, class I, C | Antigen Presentation; Interferon Response | 2.848 | 0.001147 |
| *FLT1* | Fms-related tyrosine kinase 1 | HIF1 Signaling;  VEGF Signaling | 2.578 | 0.00099 |
| *ITPR1* | Inositol 1,4,5-trisphosphate receptor, type 1 | Senescence | 2.518 | 0.000703 |
| *CDCP1* | CUB domain containing protein 1 | MAPK Signaling | 2.516 | 0.00133 |
| *PFKFB3* | 6-phosphofructo-2-kinase/ fructose-2,6-biphosphatase 3 | Glucose Metabolism; HIF1 Signaling | 2.241 | 0.000349 |
| *HLA-A* | Major histocompatibility complex, class I, A | Antigen Presentation; Interferon Response | 2.012 | 0.000122 |
| *ICAM1* | Intercellular adhesion molecule 1 | Interferon Response; NF-kB Signaling | 1.874 | 0.001445 |
| *CXXC5* | CXXC finger protein 5 | Estrogen Signaling | 1.871 | 0.000334 |
| *TLR4* | Toll-like receptor 4 | EMT; NF-kB Signaling | 1.795 | 0.004824 |
| *P4HA2* | Prolyl 4-hydroxylase, alpha polypeptide II | HIF1 Signaling | 1.704 | 0.004907 |
| *ARID4A* | AT rich interactive domain 4A (RBP1-like) | Androgen Signaling; Epigenetic & Transcriptional Regulation | 1.689 | 0.002097 |
| *ERBB3* | Erb-b2 receptor tyrosine kinase 3 | MAPK Signaling | 1.675 | 0.003634 |
| *GSK3B* | Glycogen synthase kinase 3 beta | EMT; Hedgehog; PI3K-Akt Signaling | 1.636 | 0.004628 |
| *COL4A1* | Collagen, type IV, alpha 1 | Cell Adhesion & Motility; ECM Remodeling & Metastasis; PDGF Signaling | 1.620 | 0.005894 |
| *ATOX1* | Antioxidant 1 copper chaperone | Nrf2 & Oxidative Stress | 1.594 | 0.000511 |
| *DUSP6* | Dual specificity phosphatase 6 | MAPK Signaling | 1.571 | 0.001959 |
| *PIK3R2* | Phosphoinositide-3-kinase, regulatory subunit 2 (beta) | PDGF Signaling PI3K-Akt Signaling | 1.567 | 0.003701 |
| *IFNAR2* | Interferon (alpha, beta and omega) receptor 2 | Interferon Response | 1.540 | 0.003101 |
| *CDCA5* | Cell division cycle associated 5 | Cell Cycle | -1.533 | 0.001868 |
| *BCL2L1* | BCL2-like 1 | Apoptosis; Epigenetic & Transcriptional Regulation; NF-kB Signaling | -1.546 | 0.001415 |
| *MCM2* | Minichromosome maintenance complex component 2 | Cell Cycle; mTOR Signaling | -1.565 | 0.000508 |
| *CDC25A* | Cell division cycle 25A | Cell Cycle mTOR Signaling | -1.633 | 0.002662 |
| *SKA1* | Spindle and kinetochore associated complex subunit 1 | Cell Cycle | -1.648 | 0.00315 |
| *TGM2* | Transglutaminase 2 | Hippo Signaling | -1.841 | 0.003292 |
| *SDC1* | Syndecan 1 | ECM Remodeling & Metastasis; Interleukin Signaling | -1.971 | 0.005505 |
| *SLC7A5* | Solute carrier family 7 (amino acid transporter light chain, L system), member 5 | mTOR Signaling | -2.074 | 0.005792 |
| *CAV1* | Caveolin 1, caveolae protein, 22kda | Cell Adhesion & Motility EMT | -4.193 | 0.000222 |
| *IL7R* | Interleukin 7 receptor | Interleukin Signaling | -4.508 | 0.002916 |
| *THBS1* | Thrombospondin 1 | ECM Remodeling & Metastasis; Hippo Signaling;  Myc; PDGF Signaling; TGF-beta Signaling | -4.857 | 0.00273 |

Abbreviations: Nrf2 = nuclear factor erythroid 2-related factor 2, NF-kB = nuclear factor kappa B, mTOR = mechanistic target of rapamycin kinase, TGF-beta = transforming growth factor-beta, EMT= Epithelial-to-Mesenchymal Transition, MAPK = mitogen activated protein kinase, PDGF = platelet derived growth factor, TCR = T-cell receptor, HIF1 = hypoxia-inducible factor-1, VEGF = vascular endothelial growth factor, PI3K-Akt = phosphatidylinositol 3-kinase-AKT serine-threonine protein kinase, ECM = extracellular matrix, MET = MET proto-oncogene, Myc = Myc proto-oncogene.

**Supplemental Table 3**. NanoString Human Tumor Signaling 360 Panel Analysis significantly modulated genes after CRISPR/Cas9-mediated SIRT3 KO in G361 human melanoma cells.

| Gene | Description | Molecular functions | Fold Δ | p value |
| --- | --- | --- | --- | --- |
| *KDR* | kinase insert domain receptor | VEGF Signaling | 3.777 | 0.00136 |
| *IL7* | interleukin 7 | Interleukin Signaling | 2.583 | 0.00018 |
| *UGDH* | UDP-glucose 6-dehydrogenase | Androgen Signaling | 1.903 | 6.56E-05 |
| *OAS1* | 2'-5'-oligoadenylate synthetase 1, 40/46kDa | Interferon Response | 1.856 | 0.00105 |
| *TPD52* | tumor protein D52 | Androgen Signaling | 1.740 | 5.21E-07 |
| *LDHA* | lactate dehydrogenase A | Glucose Metabolism; HIF1 Signaling; mTOR Signaling; Myc | 1.609 | 0.00035 |
| *CD44* | CD44 molecule (Indian blood group) | ECM Remodeling & Metastasis; EMT;  Immortality & Stemness; Interferon Response;  T-cell Exhaustion | 1.555 | 0.00067 |
| *EPAS1* | endothelial PAS domain protein 1 | Immortality & Stemness | 1.543 | 0.00095 |
| *ASH1L* | ash1 (absent, small, or homeotic)-like (Drosophila) | Epigenetic & Transcriptional Regulation | -1.809 | 2.22E-05 |
| *NUF2* | NUF2, NDC80 kinetochore complex component | Cell Cycle | -2.526 | 4.64E-05 |

Abbreviations: ECM = extracellular matrix, EMT= Epithelial-to-Mesenchymal Transition, VEGF = vascular endothelial growth factor, HIF1 = hypoxia-inducible factor-1, mTOR = mechanistic target of rapamycin kinase, Myc = Myc proto-oncogene

**Supplemental Table 4**. Dharmacon siSTABLE siRNA sequences for in vivo experiments.

| siRNA | Species | Sequence |
| --- | --- | --- |
| siNTC | Human & Mouse | Sense: U.A.G.C.G.A.C.U.A.A.A.C.A.C.A.U.C.A.A.U.U  Antisense: 5’- P.U.U.G.A.U.G.U.G.U.U.U.A.G.U.C.G.C.U.A.U.U |
| Custom Pool for PDX models: | | |
| siSIRT3 #1 | Human | Sense: G.C.U.U.G.A.U.G.G.A.C.C.A.G.A.C.A.A.A.U.U  Antisense: 5’-P.U.U.U.G.U.C.U.G.G.U.C.C.A.U.C.A.A.G.C.U.U |
| siSIRT3 #2 | Human | Sense: G.G.C.A.A.U.A.G.A.U.U.U.A.A.U.G.A.C.A.U.U  Antisense: 5’-P.U.G.U.C.A.U.U.A.A.A.U.C.U.A.U.U.G.C.C.U.U |
| siSIRT3 #3 | Human | Sense: G.A.C.C.A.G.A.C.A.A.A.U.A.G.G.A.U.G.A.U.U  Antisense: 5’-P.U.C.A.U.C.C.U.A.U.U.U.G.U.C.U.G.G.U.C.U.U |
| siSIRT3 #4 | Human | Sense: G.C.A.G.G.U.G.A.A.A.C.C.A.G.A.A.U.A.U.U.U  Antisense: 5’-P.A.U.A.U.U.C.U.G.G.U.U.U.C.A.C.C.U.G.C.U.U |
|  | | |
| Custom Pool for Braf^V600E^/PTEN^NULL^ model: | | |
| siSIRT3 #1 | Mouse | Sense: A.C.A.G.C.A.A.C.C.U.U.C.A.G.C.A.G.U.A.U.U  Antisense: 5’-P.U.A.C.U.G.C.U.G.A.A.G.G.U.U.G.C.U.G.U.U.U |
| siSIRT3 #2 | Mouse | Sense: C.A.A.G.G.U.U.C.C.U.A.C.U.C.C.A.U.A.U.U.U  Antisense: 5’-P.A.U.A.U.G.G.A.G.U.A.G.G.A.A.C.C.U.U.G.U.U |
| siSIRT3 #3 | Mouse | Sense: U.G.A.C.U.U.C.G.C.U.U.U.G.G.C.A.G.A.U.U.U  Antisense: 5’-P.A.U.C.U.G.C.C.A.A.A.G.C.G.A.A.G.U.C.A.U.U |
| siSIRT3 #4 | Mouse | Sense: G.A.A.C.A.U.C.G.A.C.G.G.G.C.U.U.G.A.G.U.U  Antisense: 5’-P.C.U.C.A.A.G.C.C.C.G.U.C.G.A.U.G.U.U.C.U.U |
